# Cloning, Sequencing, and Expression of Selenoprotein Transcripts in the Turkey (*Meleagris gallopavo*)

**DOI:** 10.1371/journal.pone.0129801

**Published:** 2015-06-12

**Authors:** Roger A. Sunde, Gavin R. Sunde, Colin M. Sunde, Milton L. Sunde, Jacqueline K. Evenson

**Affiliations:** 1 Department of Nutritional Sciences, University of Wisconsin, Madison, Wisconsin, United States of America; 2 Department of Animal Sciences, University of Wisconsin, Madison, Wisconsin, United States of America; University of Florida, UNITED STATES

## Abstract

The minimum Se requirement for male turkey poults is 0.3 μg Se/g – three times higher than requirements found in rodents – based on liver and gizzard glutathione peroxidase-4 (GPX4) and GPX1 activities. In addition, turkey liver GPX4 activity is 10-fold higher and GPX1 activity is 10-fold lower than in rats, and both GPX1 and GPX4 mRNA levels are dramatically down-regulated by Se deficiency. Currently, the sequences of all annotated turkey selenoprotein transcripts and proteins in the NCBI database are only “predicted.” Thus we initiated cloning and sequencing of the full turkey selenoprotein transcriptome to demonstrate expression of selenoprotein transcripts in the turkey, and to develop tools to investigate Se regulation of the full selenoproteome. Total RNA was isolated from six tissues of Se-adequate adult tom turkeys, and used to prepare reverse-transcription cDNA libraries. PCR primers were designed, based initially on chicken, rodent, porcine, bovine and human sequences and later on turkey shotgun cloning sequences. We report here the cloning of full transcript sequences for 9 selenoproteins, and 3'UTR portions for 15 additional selenoproteins, which include SECIS elements in 22 3'UTRs, and in-frame Sec (UGA) codons within coding regions of 19 selenoproteins, including 12 Sec codons in SEPP1. In addition, we sequenced the gap between two contigs from the shotgun cloning of the turkey genome, and found the missing sequence for the turkey Sec-tRNA. RTPCR was used to determine the relative transcript expression in 6 tissues. GPX3 expression was high in all tissues except kidney, GPX1 expression was high in kidney, SEPW1 expression was high in heart, gizzard and muscle, and SELU expression was high in liver. SEPP2, a selenoprotein not found in mammals, was highly expressed in liver but not in other tissues. In summary, transcripts for 24 selenoproteins are expressed in the turkey, not just predicted.

## Introduction

The minimum selenium (Se) requirement for male turkey poults is two to three times higher than requirements in rodents, chickens, and in other species when determined using selenoprotein enzyme activities as biomarkers of Se status [1–5]. In addition, turkey liver glutathione peroxidase-4 (GPX4) activity is 10-fold higher than in rats, and turkey liver GPX1 specific activity is more than 10-fold lower than in rats, showing unique regulation in at least this avian [4]. Lastly, our preliminary work indicated that both GPX1 and GPX4 mRNA levels are down-regulated by Se deficiency [4].

The Se in all selenoproteins is present as selenocysteine (Sec) in the peptide backbone, which is specified positionally by an UGA codon (normally a stop codon) in the mRNA [6]. The carbon backbone of Sec arises from serine [7] which is esterified to a unique tRNA (the selenocysteine tRNA) that recognizes the UGA codon. Inorganic Se is activated in series of novel enzyme steps to synthesize Sec while esterified to the tRNA, and then a unique stem-loop secondary structure—called a SECIS element (Sec insertion sequence)—must be present in the 3'UTR of eukaryotic selenoprotein mRNAs for Sec incorporation at the UGA position [6,8].

The first report of sequencing and annotation of the chicken genome was made in December, 2004 [9]. For humans, rodents and a number of other species, search for SECIS elements and in-frame UGA codons has resulted in the identification of the complete selenoprotein proteome (selenoproteome) [8], and the chicken selenoproteome consists of at least 23 selenoproteins [10]. Currently (February 2015) the National Center for Biotechnology Information (NCBI) annotates 23 chicken selenoprotein transcripts. Shotgun sequencing of the turkey (*Meleagris gallopavo*) genome did not identify the Sec tRNA [11] but that report indicated that selenoproteins were undoubtedly present because of our report of the GPX4 transcript [4]. Current NCBI now annotates 24 turkey selenoprotein transcripts, but all are only classified as predicted, and the corresponding proteins are all classified as predicted proteins (8 total) or as predicted low quality proteins (16 total). See S1 Table and S2 Table for the corresponding NCBI Reference Sequences and nomenclature for the turkey selenoprotein transcripts, proteins and genes, and the chicken transcripts, as of February, 2015.

Thus we initiated cloning and sequencing of the full turkey selenoprotein transcriptome to demonstrate the actual expression of selenoprotein transcripts in the turkey, to develop tools to investigate Se regulation of the full selenoproteome, and to better dissect-out the basis for differential species-based Se requirements. Here we report the cloning and sequencing of 24 selenoprotein transcripts; using these sequences, reverse-transcription polymerase chain reaction (RTPCR) primers were prepared and apparent relative expression of these selenoproteins was determined in 6 Se-adequate tissues.

## Materials and Methods

### Reagents

Molecular biology reagents were purchased from Promega (Madison, WI), Invitrogen (Carlsbad, CA) or Sigma (St. Louis, MO). All other chemicals were of molecular biology or reagent grade.

### Animals

A Nicholas white tom turkey (tom#1), fed a Se-adequate practical corn-soy-based diet (36% corn; 56% soybean meal; 28% crude protein, 2800 kcal ME/kg [12], supplemented with 0.03 mg Se/kg diet as Na_2_SeO_3_) and weighing ~30 lb (14 kg), was killed by stunning followed by exsanguination, and liver, heart, gizzard, kidney, thigh and breast tissue samples quickly removed, frozen in liquid N_2_, and stored at -80°C. This protocol was approved by the University of Wisconsin College of Agricultural and Life Sciences Animal Care and Use Committee (Protocol No. A01146). Liver, heart and gizzard tissues from a second Nicholas white-derived tom turkey (tom#2), which had been fed a proprietary practical diet, were generously donated by Jennie-O Turkey Store (Barron, WI), and were quickly removed, frozen on dry ice, and stored at -80°C.

### Enzyme Activity Assays

Tissue GPX4 activity was measured by the coupled assay procedure [13] using 78 μM phosphatidyl choline hydroperoxide (PCOOH), the GPX4-specific substrate, as described previously [4]. Total GPX activity was assayed using 120 μM H_2_O_2_, and GPX1 specific activity was calculated by subtracting the activity detected with H_2_O_2_ due to GPX4 (0.63 EU_H2O2_ /EU_PCOOH_), from the total GPX activity, as described previously [4]. Thioredoxin reductase (TXNRD) activity was assayed using 5.3 mM 5,5’-dithiobis (2-nitrobenzoic acid) (Sigma D8130), as described previously [5]. Protein concentration was determined by the method of Lowry et al [14].

### Total RNA and cDNA Libraries

Total RNA was isolated from 75–100 mg of tissue homogenized in 1 ml TRIzol Reagent (cat. #15596–026, Invitrogen), following the manufacturer’s protocol, as we have done in previous studies [5]. The RNA pellet was dissolved in diethyl pyrocarbonate (DEPC)-treated water and quantitated using a ND-1000 UV-Vis Spectrophotometer (NanoDrop Technologies, Wilmington, DE). Total RNA (1 μg) was reverse transcribed to cDNA using the RETROscript kit (AM1710, Ambion Inc., Austin, TX), following the manufacturer’s protocol. Initial cDNA libraries were prepared using the Ambion Oligo(dT) primer or a similar oligo(dT) primer (P148: TCTAGACCTCAGGTTTTTTTTTTTTTTTTTT); the Ambion Random Decamer primer was used to make subsequent cDNA libraries for larger transcripts. cDNA libraries for RTPCR were prepared using Ambion Oligo(dT) primer. Working stocks of cDNA libraries were diluted 1/50 in DEPC-treated water.

### Primers

PCR primers and RTPCR primers (see below) were typically designed as 20-mers with a dissociation temperature of ~60°C, and with a 1–2 nt G-C lock as 3'-terminal bases. Initially, forward primers for a given selenoprotein were based on chicken transcript sequences when available, or based on consensus sequences of homologous rodent, porcine, bovine and human genes, and designed to amplify an ~800 nt fragment. After the shotgun cloning of the turkey genome, predicted turkey transcript sequences were used when available. From inception, over 500 primers were designed and synthesized by the UW Biotechnology DNA Synthesis lab for this project. Initially primers were designed by hand, but most screening for primer sequences was conducted using Primer3 or Primer3Plus (http://primer3plus.com/cgi-bin/dev/primer3plus.cgi).

### Cloning

PCR cloning was conducted using 1 μl of a cDNA working (1/50) stock, 1 μl of a forward primer (10 μM) and 1 μl of oligo(dT) primer (10 μM) using reagents and protocol from the Ambion RETROscript Kit (AM1710). Alternatively, 1 μl of cDNA working stock, 1 μl each of forward and reverse primers were combined with 9.5 μl DEPC-treated water and 12.5 μl PCR MasterMix (Promega). Reaction components were mixed in 500 μl tubes on ice. To start the reaction, reactions were incubated at 95°C for >3 min to dissociate the cDNA, and then the reaction started by “hot-start” addition of Taq polymerase/PCR-Mix, and then subjected to cycles of 15 sec 95°C, followed by 1 min at 60°C for 50 cycles in a Perkin Elmer 480 Thermal Cycler. At the end of the run, samples were held at 4°C and then stored at 4°C.

Resulting amplified cDNA (3 μl of the 24–25 μl reaction) was ligated into pGEM-T vector (pGEM-T Vector System II, Promega A3610) following manufacturer’s protocol. Following overnight incubation at 4°C, 2 μl (of the 10 μl reaction) was used to transform competent JM-109 cells (50 μl, Promega L2001) following manufacturer’s protocol. Transformed cells were spread on 1.5% agar LB (Luria-Bertani media) plates containing 50 μg/ml ampicillin that were top-treated with 100 μl of 100 mM IPTG (Sigma I5502) and 20 μl of 50 mg/ml X-gal (Promega V394A) for blue-white selection, and incubated overnight at 37°C. Individual white colonies were picked and grown overnight in 5 ml cultures of LB media containing 50 μg/ml ampicillin. Resulting plasmids were isolated using polyethylene glycol precipitation, including RNase A treatment to removing contaminating RNA, and suspended in DEPC-treated water [15]. Plasmids preps were assessed by A_260_/A_280_ ratio (>1.9 and typically >1.95) and yield was quantitated at A_260_ using a UV-Vis spectrophotometer (NanoDrop). Plasmid preps were screened by restriction enzyme digestion, using the convenient BstZ1 (Promega R6881) site on both sides of the pGEM-T multiple cloning site, followed by gel electrophoresis to identify clones for sequencing with the expected or interesting insert size.

Successfully for nine genes, RNA Ligase Mediated Rapid Amplification of 5' cDNA Ends (5'RACE) was also conducted using 5'-cap-specific ligation and random decamer reverse transcription, following the manufacturer’s protocol (Ambion AM-1700). Nested 5'RACE primers and sequence-specific reverse primers were then used to clone formerly-capped transcripts.

### Sequencing

Selected plasmid inserts were sequenced in both directions by the UW Biotechnology DNA Sequencing Center, taking advantage of the T7 and SP6 sequences in the pGEM-T plasmid. Briefly, purified plasmid (1000 ng) and 20 pmole of either the T7 or SP6 primer in 24 μl DEPC-treated water were analyzed. Typical analyses provided high-quality sequence for >1000 nt in each direction. Sequences in both directions from a minimum of two independent clones were aligned using Bioedit [16] to obtain sequences that were submitted to NCBI using the Sequin submission system.

### RTPCR

Using resulting turkey selenoprotein transcript sequences, gene-specific primers were designed to span apparent splice-junctions and amplify 120–150 basepair (bp) fragments. Splice junction locations were determined by aligning transcript sequences with the species-specific NCBI genome or with whole-genome shotgun contig or with *Gallus gallus* nucleotide annotation. RTPCR primers were next screened by PCR reactions against reverse-transcribed cDNA working stocks followed by gel electrophoresis to confirm expected fragment size. Lastly, preliminary RTPCR reactions were conducted to verify that the product yielded a single dissociation (derivative) peak and consistent amplification signal.

RTPCR analyses were conducted using 96-well plates. In triplicate, the final 25 μL reactions contained 10 ng reverse transcribed RNA (10 μl of 1/50 working stock), 0.2 mmol/L gene specific forward and reverse primers, and 1X SybrGreen PCR Master Mix (#4309155, Applied Biosystems, Foster City, CA), according to manufacturer’s protocols. Reactions were followed in an ABI Prism 7000 (Applied Biosystems) with initial stages of 50°C for 2 min and 95°C for 10 min, followed by 50 cycles consisting of 95°C for 15 sec and 60°C for 2 min. A dissociation curve was run for each plate to confirm the production of a single product. The amplification efficiency for each gene was determined using the DART-PCR program [17]. The mRNA relative abundance was calculated according to Pfaffl [18], accounting for gene-specific efficiencies, and normalized relative to the mean GPX1 expression. To compare transcript expression of different selenoproteins, relative abundance was normalized for basepair length of the amplified fragment.

### Analysis

Sequences were determined in both directions for at least two independent clones. Sequences were compared to determine sequence identity using NCBI Blast software (http://blast.ncbi.nlm.nih.gov/Blast.cgi). Estimates of selenoprotein relative expression were determined in triplicate and used to calculate means and standard deviation (SD).

## Results

While we had previously partially cloned and sequenced transcripts for GPX4 and GPX1 for the turkey [4], this project began in earnest in the summer of 2009 as a project for a recent high-school graduate. Tissues were collected and frozen from an adult tom turkey (tom#1) fed a practical corn-soy diet from the UW Poultry Arlington Experiment Station. An initial set of potential primers based on available NCBI chicken selenoprotein sequences plus rodent, bovine, porcine and human sequences were designed to make an initial survey of selenoprotein expression in different tissues and as starting primers for cloning. The project was continued in summers of 2010–2013 as high-school and undergraduate projects. Preliminary partial sequences for 13 turkey selenoproteins were submitted to NCBI in early 2012; these sequences, now updated, and 12 new sequences are available from NCBI.

### Selenoenzyme Activity

Tissue GPX1 and GPX4 activities in both tom turkeys were similar to levels in 4-wk-old male turkey poults supplemented with 0.3–0.5 μg Se/g diet in our previous study [4], showing that these tom turkeys were Se-adequate (Table 1). Overall ranking indicates that kidney had the highest GPX1 specific activity, gizzard and heart had moderate activity, followed by liver, with the lowest GPX1 specific activity in thigh and breast. Similar rankings for GPX4 activity were gizzard > heart, liver > kidney > thigh, breast. Kidney had the highest TXNRD activity, followed by heart and liver.

**Table 1:** Glutathione peroxidase and thioredoxin reductase activities in turkey tissues.

	GPX1 Activity ^1^ (EU/g prot)		GPX4 Activity ^1^ (EU/g prot)		TXNRD Activity ^2^ (EU/g prot)	
Tissue	Tom#1	Tom#2	Tom#1	Tom#2	Tom#1	Tom#2
**Liver**	**42.2**	**41.7**	**45.6**	**47.5**	**4.07**	**3.43**
**Heart**	**77.2**	**59.2**	**33.6**	**27.7**	**4.38**	**3.83**
**Gizzard**	**83.1**	**77.9**	**7.9**	**8.3**	**1.31**	**2.55**
**Kidney**	**481.5**		**24.3**		**6.99**	
**Thigh**	**25.5**		**6.0**		**1.85**	
**Breast**	**9.0**		**4.0**		**0.53**	

^1^GPX1 and GPX4 activities determined on 15,000 x g supernatants of 10% homogenates, as described by Sunde and Hadley [4], and prepared from the same frozen tissues used for total RNA analysis. Gpx4 activity assayed using 78 μM PCOOH. GPX1 activity was assayed using 120 μM H_2_O_2_, and then corrected for GPX4-dependent activity, to yield the GPX1 activities shown in the table. Activity values are expressed per gram protein.

^2^TXNRD activities determined on dialyzed 15,000 x g supernatants of 10% homogenates using gold-thioglucose inhibition of 5,5’-dithiobis (2-nitrobenzoic acid) reduction, as described by Barnes et al. [5]. Activity values are expressed per gram protein.

### Selenoprotein Transcript Sequences

**Fig 1:**
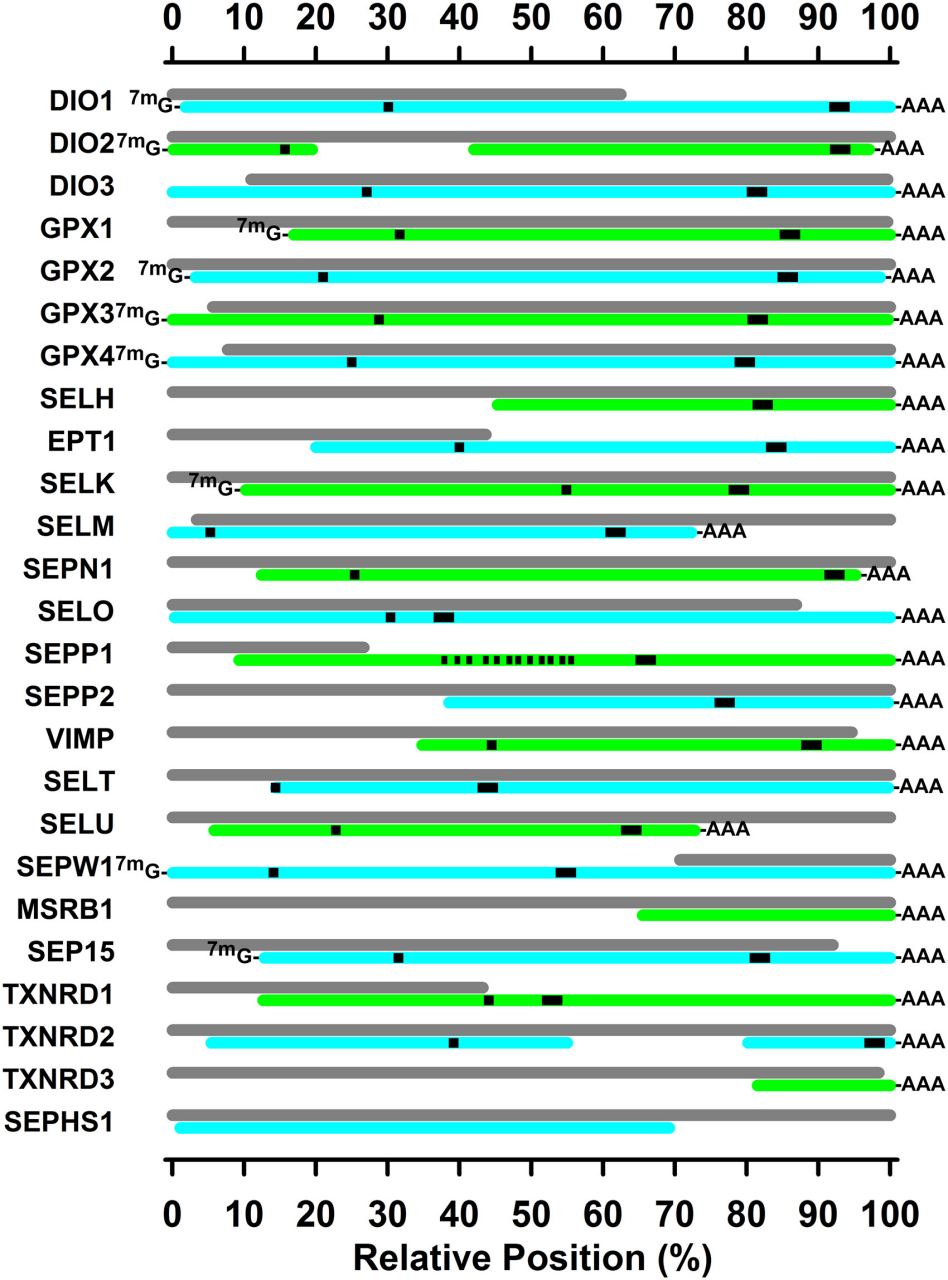
Sequenced turkey selenoprotein transcripts. Colored bars show the alignment of sequenced selenoprotein transcripts relative to the NCBI predicted turkey sequences, shown as gray bars. Sequence details are given in Table 2, and related NCBI sequences are listed in S1 Table and S2 Table. Sequences with a complete 3'UTR sequence, as determined by 3'RACE using oligo(dT) primers, are marked with-AAA. Sequences with a complete 5'UTR sequence, as determined by 5'RACE using 5'-cap-specific ligation, are marked with ^7m^G-. The position of the UGA codon is indicated by a black square, and the position of the SECIS element is indicated by a black rectangle. For SEPP2, black vertical lines indicate the relative position of the 12 UGA codons.

Fig 1 summarizes the cloning of turkey selenoprotein transcripts, and Table 2 provides details. We have cloned and sequenced portions of 24 selenoproteins from the turkey. This includes full transcript sequences for 9 selenoproteins, and 3'UTR portions for 15 additional selenoproteins. Furthermore, these 3'UTR sequences include sequences for 22 SECIS elements that were identified using the SECISearch web-based programs [19]. Lastly, the coding sequences identified TGA (UGA) in-frame Sec codons within 19 selenoproteins, including 12 Sec codons in SEPP1. We could identify only one SECIS element in the turkey SEPP1 3’UTR, whereas mammalian SEPP1 (with 10 UGAs) has two SECIS elements [6]. Clearly, transcripts for 24 selenoproteins are expressed in the turkey, not just predicted.

**Table 2:** Sequenced turkey selenoprotein transcript details.

		NCBI	Sequence^1^			Transcript Position^2^			Protein Position^3^	
Gene	Alias	Acc. No.	5'	BP	3'	UGA	SECIS	Coding	AA	Cys
GPX1		GQ502186	Y	879	Y	152–154	701–739	38–625	195	39
GPX2		KR528572	Y	769	Y	139–141	639–680	19–594	191	41
GPX3		JQ660508	Y	1001	Y	283–285	795–842	73–732	219	71
GPX4		GQ502187	Y	833	Y	205–207	648–693	4–582	192	68
DIO1		JQ945764	Y	1397	Y	398–400	1270–1310	29–769	246	124
DIO2		KR528570	Y	1213>		974–976		581–1213>	211>	132
				<3451	Y		6592–6638			
DIO3		KR528571	N	<1358	Y	359–361	1069–1121	<1–772	<256	120
SELH	C5H11orf31	KR528574	N	<389	Y	na	250–299	<1–178	<58	na
EPT1	SELI	JQ616821	N	<2339	Y	578–580	1839–1886	<1–631	<209	193
SELK	LOC100544511	JQ616819	Y	604	Y	294–296	447–491	18–305	95	93
SELM	LOC100546114	JQ660505	N	<965	Y	45–47	800–840	<1–395	<130	15
SELO	LOC100539640	KR528575	N	<4848	Y	1459–1461	1786–1833	<1–1470	<489	487
VIMP	SELS	JQ422051	N	<845	Y	124–126	680–727	<1–132	<43	42
SELT	LOC100548711	JQ670267	N	<1070	Y	na	349–394	<1–281	92	na
SELU	FAM213A	KR528576	N	1059	Y	240–242	888–924	<674	223	80
MSRB1	SEPX1	KR528573	N	<525	Y	na	na	na	na	na
SEPHS1		KR528577	N	<2280>	N	na	na	55–1233	392	na
SEP15		JQ945765	Y	1372	Y	288–290	1053–1096	18–500	160	91
SEPN1		JQ670266	N	<3619	Y	566–568	3444–3485	<1–955	317	189
SEPP1		JQ660509	N	<2141	Y	677–679, 716–718, 755–757, 809–811, 845–847, 884–886, 890–892, 911–913, 953–955, 959–961, 980–982, 986–988	1313–1357	<1–1000	<332	226, 239, 252, 270, 282, 295, 297, 304, 318, 320, 327, 329
SEPP2		JQ945763	N	<936	Y		566–614	<329	<108	na
SEPW1		JQ436639	Y	791	Y	109–111	419–466	73–330	85	13
TXNRD1		KR528578	N	<2842	Y	1019–1021	1282–1329	<1–1027	<341	340
TXNRD2		KR528579	N	<1975>	Y	1346–1348		<1–1354	<450	449
				<802			3652–3698			
TXNRD3		KR528580	N	<552	Y	na	na	na	na	na

^1^For each selenoprotein transcript, the sequenced base pairs (bp) are specified, with “Y” indicating that a complete 5'UTR was sequenced as determined by 5'RACE using 5'-cap-specific ligation, and a complete 3'UTR was sequenced sequence as determined by 3'RACE using oligo(dT) primers. Missing 5’UTR is indicated with <, and missing 3’UTR is indicated with >.

^2^For each sequenced transcript, the position of the UGA codon, SECIS element, and coding region (CDS) is specified. Missing 5’ coding sequence is indicated with <, and missing 3’ coding sequence is indicated with >.

^3^ For each sequenced coding sequence, the number of corresponding amino acid (AA) residues is specified along with the position of the selenocysteine (Sec). Missing N-terminal amino acids are indicated with <, and missing C-terminal amino acids are indicated with >.

### Sec tRNA

**Fig 2:**
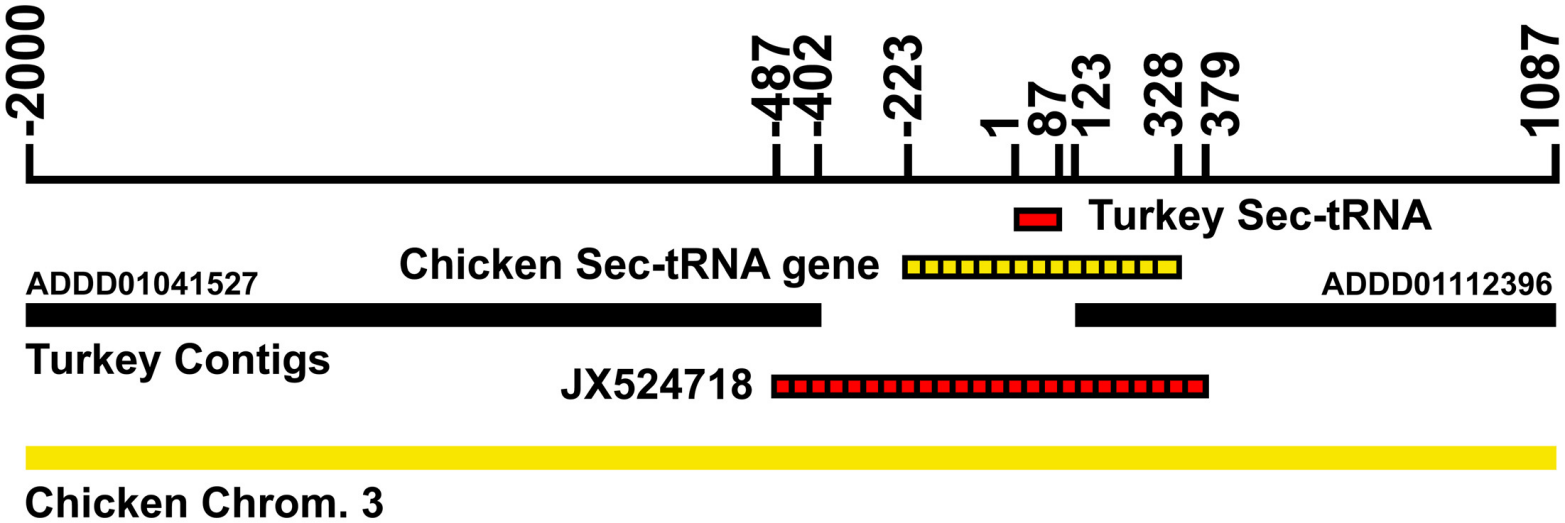
Cloned turkey Sec-tRNA alignment with turkey chromosome 3 shotgun contigs and chicken chromosome 3. The DNA sequence for the 87 nt turkey Sec-tRNA (red bar) lies between contig ADDD01041537 and ADD01112396 (black bars) in a 525 bp gap. PCR cloning and sequencing across this gap revealed a 866 bp sequence (JX524718, hatched red bar, bp -487 to 379) that aligns with the chicken Sec-tRNA gene (bp -223 to 328 in the figure, hatched yellow bar), including 100% sequence identity with the chicken Sec-tRNA sequence. Yellow bar represents chicken chromosome 3.

The September 2010 report of the next-generational (shotgun) cloning of the turkey genome was unable to locate Sec tRNA sequence [11], but the authors suggested this was “most likely an artifact of incomplete data rather than a true loss, given the presence of likely genes for selenoproteins such as GPX4,” citing our report [4]. Alignment of the portion of chicken chromosome 3, which contains the chicken Sec tRNA gene (GenBank: K01941.1) [20], with contigs from shotgun sequencing revealed that the turkey Sec-tRNA gene likely resided in the gap between contigs ADDD01041527 and ADDD01112396 on turkey chromosome 3. Using total DNA isolated from turkey liver and primers based on the chicken Sec tRNA [20] and in these two contigs, we PCR cloned and sequenced the gap (Fig 2). The 866 bp gap (tRNAu1, NCBI GenBank: JX524718.1) contained a 551 bp sequence that aligned with the 543 nt chicken Sec-tRNA gene (83% identify, including 100% identity with the 87 nt chicken tRNA sequence.

### Selenoproteins and Sec

These sequenced selenoprotein coding regions encode the apparent full coding region for 9 selenoproteins and portions of the coding region for 13 additional selenoproteins (Table 2). This includes Sec in 19 selenoproteins (same as the number of in-frame TGA Sec codons), and including 12 Sec amino acids in SEPP1. A 13^th^ Sec is apparently 11 amino acid residues upstream of the peptide specified by our cloned SEPP1 transcript according to the predicted SEPP1 transcript sequence.

SEPHS2 is the Sec-containing selenophosphate synthetase present in mammals, and is thought to be primary selenophosphate synthetase used for Sec synthesis [21]. Despite designing multiple primers for SEPHS2, we were unable to clone any sequences for SEPP2 in the turkey. In addition, we have not been able to clone any of transcript sequences for SELV or GPX6. Thus it appears that these three selenoprotein genes, found in mammals, may not be present in the turkey. We were able to partially clone turkey SEPHS1 (Fig 1, Table 2), however, including sequences encoding 392 amino acids, providing strong evidence for expression of this crucial member of the novel Sec synthesis pathway.

### RTPCR Selenoprotein Primers

Using our selenoprotein transcript sequences as well as NCBI sequences, we designed and tested RTPCR primers for these identified 24 turkey selenoproteins plus SEPHS1, GAPDH and ACTB (Table 3). Splice junction locations were determined by aligning transcript sequences with the species-specific NCBI genome or with whole-genome shotgun contig or with *Gallus gallus* nucleotide annotation. Gene transcript-specific primers were then designed to span apparent splice-junction and amplify 120–150 base fragments. RTPCR primers were next screened by PCR reactions against reverse-transcribed cDNA working stocks followed by gel electrophoresis to confirm expected fragment size (data not shown). Lastly, preliminary RTPCR reactions verified that the product yielded a single dissociation (derivative) peak and consistent amplification (data not shown).

**Table 3:** qRT-PCR primers for turkey selenoproteins^1^.

Gene	Forward Primer	Reverse Primer	Fragment ^2^ bp)
DIO1	tttggaagctgcacctgacc	aaggcccatccatctactgc	134
DIO2	ggaacagcttcctcctggac	accaccaacactcttccagc	125
DIO3	ctacttcgagcggctctacg	tctggagccgggttttgtac	125
GPX1	gcttcccctgcaaccaattc	tcacctcgcacttctcgaac	129
GPX2	ttcccctgcaaccagttcg	tcacctggcacttctggaac	127
GPX3	tcgtccccaacttccaactc	gtggctcccagaagaggttc	138
GPX4	gatgaaggagcagcccaagg	cacggaaggatcctccatgg	127
SELH	ctcggggaacttcagcttgc	ctggccgtagagatcaaccc	135
EPT1	ttggaaccgcctttgctaac	aaccagacaccaccacgaag	126
SELK	agaggctacacatcttcctcc	tcatccacctccagccattg	135
SELM	ctcgtgctgcttagcttcag	ggggcaaactggaactcctc	152
SEPN1	cctggcagcaggaaatctcc	acagcaggattgagtgcacc	147
SELO	gcttttgccatggtgtgctg	tgtaagcatagcgccctgtg	141
SEPP1	cagagagccaaaggtgccag	tccaatctggaagcctgcag	141
SEPP2	cctacagcttcctgcacctg	ccatggtactgttggcctcc	120
VIMP	caagaagcccttgcgaggag	gtaagctgaagggaccccag	134
SELT	cagtgtatgtcaacgggtgc	tccatgtgcacattgagcttc	140
SELU	ctgtccaagctgggtgttcc	catttttcgtttgcgtgggc	141
SEPW1	gagcagccaaaaagcccac	gtggacagcatgtgccaaac	149
MSRB1	ttcaaggaccacttcgagcc	gtggatggtctcggtgaagg	120
SEP15	attgggcaggttccctcaag	gcaatgttcccactgtcgtc	132
SEPHS1	gcagcagaagaagcaggaac	tctcctggcactgcattgtc	134
TXNRD1	agaagtcacgcaaggctttg	caccagaacgcttggtgatg	131
TXNRD2	ctggctcggcgtctttttg	gatccataacactgcacagcc	129
TXNRD3	cccaaggttttgcagctgc	ccgttgagtgatgtcttgtcc	140
ACTB	agaagatctggcaccacacc	ctggggtgttgaaggtctcg	144
GAPDH	aggtgctgcccagaacatc	gcaggtcaggtcaacaacag	142

^1^Primers used for qRT-PCR based on sequenced selenoprotein transcripts, and NCBI sequences. Sequences are written 5’ to 3’.

^2^Resulting PCR fragment as predicted by transcript sequences and verified by PCR followed by gel electrophoresis.

### Relative Transcript Expression

**Fig 3:**
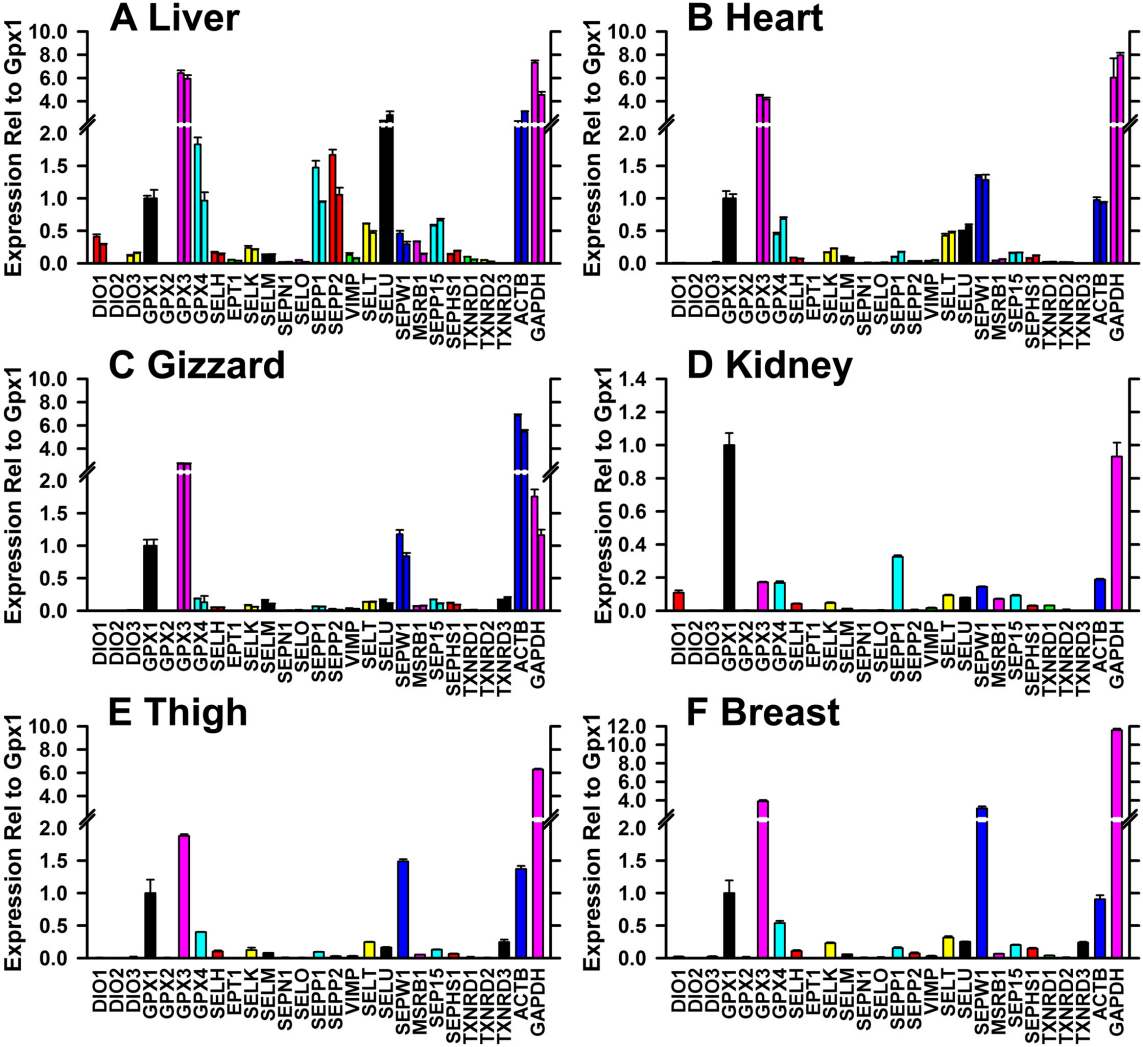
Relative expression of selenoprotein transcripts in six turkey tissues. Relative transcript expression was determined by RTPCR relative to the level of GPX1 transcript, as described in the text, in triplicate. The primer pairs used for these analyses are shown in Table 3. Double bars show the mean ± SD for both tom turkeys in liver (A), heart (B), and gizzard (C), and single bars show these values for kidney (D), thigh (E) and breast (F).

Using cDNA libraries reverse transcribed using the oligo(dT) primer, RTPCR was conducted in triplicate for liver, heart and kidney from the two tom turkeys, and in triplicate for kidney, thigh and breast from one turkey. In each tissue, transcript expression was expressed relative to the mean level of GPX1 transcript. Relative expression levels in duplicate tissues were similar (Fig 3). In liver, GPX3 and SELU transcripts were most highly expressed, at 6 and 3 times the level of GPX1 transcript. GPX4, SEPP1 and SEPP2 levels were similar to GPX1, whereas DIO2, GPX2, and TXNRD3 expression appeared to be virtually lacking (<1% of GPX1 levels) in liver. In heart, GPX3 and SEPW1 expression was 4 and 1.5 times GPX1 expression, and GPX4, SELT, and SELU expression was 50% of GPX1 levels. In gizzard, the tissue first affected by Se-deficiency, GPX3 was again highly expressed at 2.5 times GPX1 levels, and SEPW1 expression was similar to GPX1 transcript expression, whereas GPX4, SELM, SELT, SELU, SEP15 and TXNRD3 were expressed at 10–20% of GPX1 expression levels. In kidney, GPX1 transcripts were expressed at levels comparable to GAPDH expression; SEPP1 expression was next highest at 30% of GPX1 levels, and GPX3, GPX4, and SEPW1, and DIO1 expression was at 10–20% of GPX1 expression levels. The transcript expression pattern in thigh and breast was similar to gizzard with high GPX3 and SEPW1 expression.

**Fig 4:**
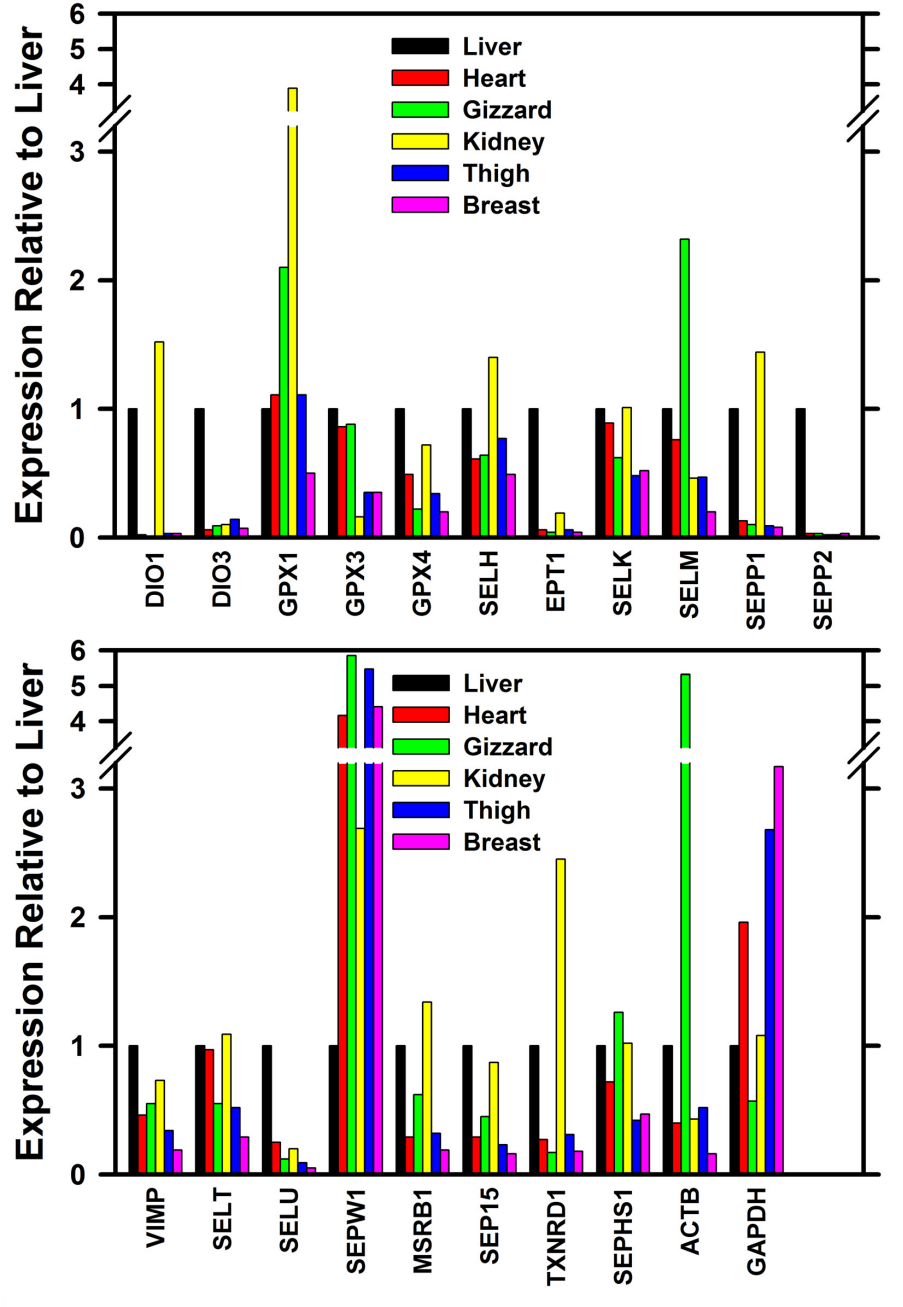
Relative tissue expression of selenoprotein transcripts. Relative transcript expression was determined by RTPCR relative to the level of selenoprotein transcript in the liver, as described in the text, in triplicate. The primer pairs used for these analyses are shown in Table 3. For each selenoprotein transcript, the expression is shown relative to liver in order of heart, gizzard, kidney, thigh, and breast.

Tissue to tissue apparent relative expression for the more abundantly expressed transcripts is shown in Fig 4. Relative to liver, GPX1 expression in kidney and gizzard was 4 and 2 time expression levels in liver, respectively, whereas GPX3 expression in kidney was <20% of the liver expression levels. GPX4 expression in gizzard was 20% of liver expression. SELU expression in liver was 5 times higher than in the other 5 tissues, whereas SEPW1 expression was 2–6 times higher in the other tissues as compared to liver. SEPP1 expression in kidney was 1.4 times higher than in liver, whereas SEPP1 expression in the other 4 tissues was #10% of expression in liver. In contrast, high SEPP2 expression appeared to be restricted to liver.

## Discussion

Current NCBI annotation of the shotgun cloning of the turkey genome includes genes and nucleotide sequences for 24 selenoproteins—all classified as “predicted.” The project reported here was able to clone and sequence 24 selenoprotein transcripts from the turkey, showing that these genes are actually expressed in the turkey. This includes full transcript sequences for 9 selenoproteins plus substantial portions including the 3'UTR terminus of the remaining 15 selenoproteins. In addition, while the Sec-tRNA sequence is not included in the shotgun cloning contigs, we found and sequenced the Sec-tRNA gene (tRNAu1), which lies in the gap between two contigs. Collectively, this shows the physical presence and expression of these predicted selenoprotein genes.

Using the same approach as used for the 24 confirmed selenoprotein transcripts, however, we were unable to clone sequences for three selenoproteins expressed in mammals. We did clone and sequence the transcript for SEPHS1, the selenoprotein synthetase that does not contain Sec, but we have been unable to clone sequences with homology to SEPHS2, the Sec-containing selenocysteine synthetase thought to have the major role in selenoprotein translation in mammals [21]. Similarly, we have been unable to clone and sequence transcripts for SELV and GPX6. In contrast, we did find two novel selenoproteins that are expressed in avians. A second selenoprotein-P that is not found in mammals, SEPP2, was highly expressed in liver and contained a SECIS element in the 3'UTR. In addition, SELU is a cysteine-containing protein in mammals [22], whereas this highly expressed transcript in the turkey has a Sec codon at the position of the cysteine codon in mammals, and has a SECIS sequence in the 3'UTR, indicating it is a selenoprotein. Thus at present, the full turkey selenoproteome appears to consist of 24 selenoproteins.

The resulting turkey selenoprotein transcripts have high sequence identify with the chicken (S3 Table). Using NCBI BLAST analysis (http://blast.ncbi.nlm.nih.gov/Blast.cgi), the turkey transcript CDS sequences averaged 96% identity with homologous chicken sequences (range 92–99%), whereas comparable sequence identities for human and mouse averaged 76 and 75%, respectively, and were as low as 67% for SEPW1 and as high as 90% for SELT. The very high sequence identity with the chicken suggests that microarray and similar applications developed for the chicken transcriptome potentially might be used successfully for the turkey and other avian species.

The predicted selenoprotein sequences in the NCBI database are predicted by automated computational analysis derived from genomic sequences. As shown in Fig 1, these predicted sequences often match well with our sequenced transcripts, but several do not include the SECIS element (DIO1, EPT1, SEPP1, SEPW1, TXNRD1), or cover the full transcript necessary to encode the full protein. More subtly, the use of 5'RACE and 3'RACE better defined the ends of the expressed transcripts in the turkey, which differ substantially from the current “predicted” transcripts for DIO1, SELM, SELO, SEPP1, SELU, and SEP15 on the 3'UTR end, and for GPX1, GPX3, GPX4, SELK, and SEP15 on the 5'UTR end. This further illustrates the value of these expressed selenoprotein transcripts to provide experimental evidence to refine these predicted annotations.

Alignment of the sequences reported here against the predicted turkey sequences, using NCBI BLAST analysis, showed 80% alignment with the currently predicted NCBI sequences (32,768 vs. 40,855 nt), with a sequence identity of 99.6% (4 differences/1000 nt), and with 1 gap/1000 nt (data not shown). Thus at the nucleotide level, the sequences determined for our birds agree highly with the sequences determined by shotgun cloning [11]. The discrepancies are due primarily to identification of the full coding region, and the UGA codon, SECIS element, and the 5'UTR and 3'UTR.

These sequences allow the design of primer pairs for these 24 selenoproteins for RTPCR analysis. The resulting expression profiles in six tissues showed good agreement using independent cDNA libraries from two adult tom turkeys for liver, heart, and gizzard. Furthermore, the selenoprotein expression profiles showed differential expression in different tissues, further substantiating the selenoprotein specificity of these primer pairs. The apparent highest GPX1 transcript expression was in kidney, the tissue with the highest measured specific GPX1 enzyme activity (Table 1). Similarly, liver, kidney and heart had the highest GPX4 transcript activity, paralleling the levels of measured GPX4 activity in these tissues. Notably, GPX3 expression was the selenoprotein with the highest expression in liver, heart and kidney, but low expression in kidney, whereas GPX3 transcript expression is high in rodent and human kidney, the tissue that is the major source of circulating GPX3 protein [23]. Kidney, the tissue with the highest TXNRD activity also had the highest TXNRD1 transcript expression. These selenoprotein transcript expression profiles clearly show distinct differences with selenoprotein expression profiles in other species.

In chickens, several studies have been conducted that examined the relative expression of selenoprotein transcripts, primarily with a focus on the impact of Se deficiency, but also with data on expression in Se-adequate chicks [24–26]. We found in turkey liver that GPX3 mRNA was most highly expressed, followed by SELU, GPX4, SEPP2, and SEPP1, and in muscle tissues we found that GPX3 mRNA was also most highly expressed, followed by SEPW1 and GPX1 (Fig 3). In Se-supplemented chicks at day 25, Liu et al. [24] also found these transcripts were highly expressed in liver, and Yao et al. [25] found in that transcripts for GPX3 and SEPW1, but not GPX1, were highly expressed in pectoral muscle at day 42. Huang et al. [26] also reported that SEPW1 was highly expressed in Se-adequate chicken muscle at day 42.

Collectively, these 24 selenoproteins sequences and resulting RTPCR primer pairs will be useful tools for assessment of the Se regulation of selenoprotein expression in the turkey, and that may be useful as molecular biomarkers for assessment of Se status and Se requirements, as we have done in rodents [5]. Specifically, their use may allow us to better compare Se requirements in different species, and to dissect-out the basis for differences in Se requirements. In addition, they may help to generate hypotheses as to why certain tissues are the target tissues for development of Se-deficiency disease.

## Supporting Information

S1 Table NCBI Reference Sequences and Nomenclature for Turkey Selenoprotein Transcripts and Selenoproteins. For each turkey selenoprotein transcript and selenoprotein, the NCBI reference sequence number and descriptive nomenclature provided (compiled as of February, 2015).
(PDF)

S2 Table NCBI Reference Sequences and Nomenclature for Chicken Selenoprotein Transcripts and Turkey Selenoprotein Genes. For each selenoprotein, the NCBI reference sequence number and descriptive nomenclature provided for the chicken selenoprotein transcripts and turkey selenoprotein genes (compiled as of February, 2015).
(PDF)

S3 Table Sequence Identity of Turkey Selenoprotein Transcripts relative to Chicken, Human, and Mouse sequences. For each selenoprotein, transcript coding sequences (CDS) in the transcript sequences reported here were compared with coding sequences for chicken (*Gallus gallus*), human (*Homo sapiens*), and mouse (*Mus musculus*) using NCBI Blast software (http://blast.ncbi.nlm.nih.gov/Blast.cgi), to determine sequence identity.
(PDF)
