# Supplementary material for: Cloning, Sequencing, and Expression of Selenoprotein Transcripts in the Turkey (Meleagris gallopavo)
Source: PLoS One. 2015 Jun 12;10(6):e0129801. doi: 10.1371/journal.pone.0129801 (PMC4466519; doi:10.1371/journal.pone.0129801)
Supplement: S1 Table — For each turkey selenoprotein transcript and selenoprotein, the NCBI reference sequence number and descriptive nomenclature provided (compiled as of February, 2015). (PDF) [file pone.0129801.s001.pdf]

S1 Table. NCBI Reference Sequences and Nomenclature for Turkey Selenoprotein Transcripts and Selenoproteins, page 1

Gene	Turkey Transcript No	Turkey Transcript Nomemclature	Turkey Protein No	Turkey Protein Nomemclature
**GPX1**	XM_003210002.2	PREDICTED: Meleagris gallopavo glutathione peroxidase 1 (GPX1), mRNA	XP_003210050.2	PREDICTED: LOW QUALITY PROTEIN: glutathione peroxidase 1 [Meleagris gallopavo]
**GPX2**	XM_010710801.1	PREDICTED: Meleagris gallopavo glutathione peroxidase 2 (gastrointestinal) (GPX2), mRNA	XP_010709103.1	PREDICTED: LOW QUALITY PROTEIN: glutathione peroxidase 2 [Meleagris gallopavo]
**GPX3**	XM_010719196.1	PREDICTED: Meleagris gallopavo glutathione peroxidase 3 (plasma) (GPX3), mRNA	XP_010717498.1	PREDICTED: LOW QUALITY PROTEIN: glutathione peroxidase 3 [Meleagris gallopavo]
**GPX4**	XM_010724964.1	PREDICTED: Meleagris gallopavo glutathione peroxidase 4 (GPX4), partial mRNA	XP_010723266.1	PREDICTED: LOW QUALITY PROTEIN: phospholipid hydroperoxide glutathione peroxidase, mitochondrial, partial [Meleagris gallopavo]
**DIO1**	XM_003208875.2	PREDICTED: Meleagris gallopavo deiodinase, iodothyronine, type I (DIO1), mRNA	XP_003208923.1	PREDICTED: LOW QUALITY PROTEIN: type I iodothyronine deiodinase [Meleagris gallopavo]
**DIO2**	XM_003206645.2	PREDICTED: Meleagris gallopavo deiodinase, iodothyronine, type II (DIO2), mRNA	XP_003206693.2	PREDICTED: LOW QUALITY PROTEIN: type II iodothyronine deiodinase [Meleagris gallopavo]
**DIO3**	XM_010712037.1	PREDICTED: Meleagris gallopavo deiodinase, iodothyronine, type III (DIO3), partial mRNA	XP_010710339.1	PREDICTED: LOW QUALITY PROTEIN: type III iodothyronine deiodinase, partial [Meleagris gallopavo]
**SELH C5H11orf31**	XM_010711191.1	PREDICTED: Meleagris gallopavo chromosome 5 open reading frame, human C11orf31 (C5H11orf31), mRNA	XP_010709493.1	PREDICTED: LOW QUALITY PROTEIN: selenoprotein H [Meleagris gallopavo]
**EPT1 SELI**	XM_003204565.2	PREDICTED: Meleagris gallopavo ethanolaminephosphotransferase 1 (CDP-ethanolaminespecific) (EPT1), mRNA	XP_003204613.2	PREDICTED: ethanolaminephosphotransferase 1 [Meleagris gallopavo]

Compiled as of February, 2015

S1 Table. NCBI Reference Sequences and Nomenclature for Turkey Selenoprotein Transcripts and Selenoproteins, page 1

Gene	Turkey Transcript No	Turkey Transcript Nomemclature	Turkey Protein No	Turkey Protein Nomemclature
**SELK LOC100544511**	XM_003210062.2	PREDICTED: Meleagris gallopavo selenoprotein K (LOC100544511), mRNA	XP_003210110.1	PREDICTED: selenoprotein K [Meleagris gallopavo]
**SELM LOC100546114**	XM_010720450.1	PREDICTED: Meleagris gallopavo selenoprotein M (LOC100546114), partial mRNA	XP_010718752.1	PREDICTED: LOW QUALITY PROTEIN: selenoprotein M, partial [Meleagris gallopavo]
**SELO LOC100539640**	XM_010706172.1	PREDICTED: Meleagris gallopavo selenoprotein O (LOC100539640), mRNA	XP_010704474.1	PREDICTED: selenoprotein O [Meleagris gallopavo]
**VIMP SELS**	XM_010717651.1	PREDICTED: Meleagris gallopavo VCP-interacting membrane protein (VIMP), partial mRNA	XP_010715953.1	PREDICTED: selenoprotein S, partial [Meleagris gallopavo]
**SELT LOC100548711**	XM_010717099.1	PREDICTED: Meleagris gallopavo selenoprotein T (LOC100548711), mRNA	XP_010715401.1	PREDICTED: selenoprotein T [Meleagris gallopavo]
**SELU FAM213A**	XM_010714114.1	PREDICTED: Meleagris gallopavo family with sequence similarity 213, member A (FAM213A), mRNA	XP_010712416.1	PREDICTED: LOW QUALITY PROTEIN: redox-regulatory protein FAM213A [Meleagris gallopavo]
**MSRB1 SEPX1**	XM_010719532.1	PREDICTED: Meleagris gallopavo methionine sulfoxide reductase B1 (MSRB1), mRNA	XP_010717834.1	PREDICTED: methionine-R-sulfoxide reductase B1 [Meleagris gallopavo]
**SEP15**	XM_010715957.1	PREDICTED: Meleagris gallopavo 15 kDa selenoprotein (LOC100547530), mRNA	XP_010714259.1	PREDICTED: LOW QUALITY PROTEIN: 15 kDa selenoprotein [Meleagris gallopavo]
**SEPN1**	XM_010723449.1	PREDICTED: Meleagris gallopavo selenoprotein N, 1 (SEPN1), mRNA	XP_010721751.1	PREDICTED: LOW QUALITY PROTEIN: selenoprotein N [Meleagris gallopavo]
**SEPP1**	XM_010725387.1	PREDICTED: Meleagris gallopavo selenoprotein P, plasma, 1 (SEPP1), mRNA	XP_010723689.1	PREDICTED: LOW QUALITY PROTEIN: selenoprotein P [Meleagris gallopavo]

Compiled as of February, 2015

S1 Table. NCBI Reference Sequences and Nomenclature for Turkey Selenoprotein Transcripts and Selenoproteins, page 1

Gene	Turkey Transcript No	Turkey Transcript Nomemclature	Turkey Protein No	Turkey Protein Nomemclature
**SEPP2**	XM_010716185.1	PREDICTED: Meleagris gallopavo selenoprotein Pb-like (LOC100546913), mRNA	XP_010714487.1	PREDICTED: LOW QUALITY PROTEIN: selenoprotein Pb-like [Meleagris gallopavo]
**SEPW1**	XR_796429.1	PREDICTED: Meleagris gallopavo uncharacterized LOC104915679 (LOC104915679), transcript variant X1, ncRNA		
**TXNRD1**	XM_010712365.1	PREDICTED: Meleagris gallopavo thioredoxin reductase 1 (TXNRD1), mRNA	XP_010710667.1	PREDICTED: thioredoxin reductase 1, cytoplasmic [Meleagris gallopavo]
**TXNRD2**	XM_003210867.2	PREDICTED: Meleagris gallopavo thioredoxin reductase 2 (TXNRD2), mRNA	XP_003210915.2	PREDICTED: thioredoxin reductase 2, mitochondrial [Meleagris gallopavo]
**TXNRD3**	XM_010718544.1	PREDICTED: Meleagris gallopavo thioredoxin reductase 3 (TXNRD3), mRNA	XP_010716846.1	PREDICTED: thioredoxin reductase 3 [Meleagris gallopavo]
**SEPHS1**	XM_010717360.1	PREDICTED: Meleagris gallopavo selenophosphate synthetase 1 (SEPHS1), mRNA	XP_010715662.1	PREDICTED: selenide, water dikinase 1 [Meleagris gallopavo]

Compiled as of February, 2015
