# Supplementary material for: Cloning, Sequencing, and Expression of Selenoprotein Transcripts in the Turkey (Meleagris gallopavo)
Source: PLoS One. 2015 Jun 12;10(6):e0129801. doi: 10.1371/journal.pone.0129801 (PMC4466519; doi:10.1371/journal.pone.0129801)
Supplement: S2 Table — For each selenoprotein, the NCBI reference sequence number and descriptive nomenclature provided for the chicken selenoprotein transcripts and turkey selenoprotein genes (compiled as of February, 2015). (PDF) [file pone.0129801.s002.pdf]

S2 Table. NCBI Reference Sequences and Nomenclature for Chicken Selenoprotein Transcripts and Turkey Selenoprotein Genes, page 1

Gene	Chicken Transcript No	Chicken Transcript Nomemclature	Turkey Gene No	Turkey Gene Nomemclature
**GPX1**	NM_001277853.1	Gallus gallus glutathione peroxidase 1 (GPX1), mRNA	NC_015024.2	Meleagris gallopavo isolate NT-WF06-2002-E0010 breed Aviagen turkey brand Nicholas breeding stock chromosome 14, Turkey_5.0, whole genome shotgun sequence
**GPX2**	NM_001277854	Gallus gallus glutathione peroxidase 2 (gastrointestinal) (GPX2), mRNA	NC_015015.2	Meleagris gallopavo isolate NT-WF06-2002-E0010 breed Aviagen turkey brand Nicholas breeding stock chromosome 5, Turkey_5.0, whole genome shotgun sequence
**GPX3**	NM_001163232.2	Gallus gallus glutathione peroxidase 3 (GPX3), mRNA	NC_015025.2	Meleagris gallopavo isolate NT-WF06-2002-E0010 breed Aviagen turkey brand Nicholas breeding stock chromosome 15, Turkey_5.0, whole genome shotgun sequence
**GPX4**	XM_003642871.2	PREDICTED: Gallus gallus glutathione peroxidase 4 (GPX4), mRNA	NC_015040.2	Meleagris gallopavo isolate NT-WF06-2002-E0010 breed Aviagen turkey brand Nicholas breeding stock chromosome 30, Turkey_5.0, whole genome shotgun sequence
**DIO1**	NM_001097614.1	Gallus gallus deiodinase, iodothyronine, type I (DIO1), mRNA	NC_015020.2	Meleagris gallopavo isolate NT-WF06-2002-E0010 breed Aviagen turkey brand Nicholas breeding stock chromosome 10, Turkey_5.0, whole genome shotgun sequence
**DIO2**	NM_204114.3	Gallus gallus deiodinase, iodothyronine, type II (DIO2), mRNA	NC_015015.2	Meleagris gallopavo isolate NT-WF06-2002-E0010 breed Aviagen turkey brand Nicholas breeding stock chromosome 5, Turkey_5.0, whole genome shotgun sequence

Compiled as of February, 2015

S2 Table. NCBI Reference Sequences and Nomenclature for Chicken Selenoprotein Transcripts and Turkey Selenoprotein Genes, page 1

Gene	Chicken Transcript No	Chicken Transcript Nomemclature	Turkey Gene No	Turkey Gene Nomemclature
**DIO3**	NM_001122648.1	Gallus gallus deiodinase, iodothyronine, type III (DIO3), mRNA	NC_015015.2	Meleagris gallopavo isolate NT-WF06-2002-E0010 breed Aviagen turkey brand Nicholas breeding stock chromosome 5, Turkey_5.0, whole genome shotgun sequence
**SELH C5H11orf31**	NM_001277865.1	Gallus gallus selenoprotein H-like (LOC100858006), mRNA	NC_015015.2	Meleagris gallopavo isolate NT-WF06-2002-E0010 breed Aviagen turkey brand Nicholas breeding stock chromosome 5, Turkey_5.0, whole genome shotgun sequence
**EPT1 SELI**	NM_001031528.2	Gallus gallus ethanolaminephosphotransferase 1 (CDP-ethanolamine-specific) (EPT1), mRNA	NC_015012.2	Meleagris gallopavo isolate NT-WF06-2002-E0010 breed Aviagen turkey brand Nicholas breeding stock chromosome 2, Turkey_5.0, whole genome shotgun sequence
**SELK LOC100544511**	NM_001025441.2	Gallus gallus selenoprotein K (SELK), mRNA	NC_015024.2	Meleagris gallopavo isolate NT-WF06-2002-E0010 breed Aviagen turkey brand Nicholas breeding stock chromosome 14, Turkey_5.0, whole genome shotgun sequence
**SELM LOC100546114**	NM_001277859.1	Gallus gallus selenoprotein M (SELM), mRNA	NC_015027.2	Meleagris gallopavo isolate NT-WF06-2002-E0010 breed Aviagen turkey brand Nicholas breeding stock chromosome 17, Turkey_5.0, whole genome shotgun sequence
**SELO LOC100539640**	NM_001115017.1	Gallus gallus selenoprotein O (SELO), mRNA	NC_015011.2	Meleagris gallopavo isolate NT-WF06-2002-E0010 breed Aviagen turkey brand Nicholas breeding stock chromosome 1, Turkey_5.0, whole genome shotgun sequence

Compiled as of February, 2015

S2 Table. NCBI Reference Sequences and Nomenclature for Chicken Selenoprotein Transcripts and Turkey Selenoprotein Genes, page 1

Gene	Chicken Transcript No	Chicken Transcript Nomemclature	Turkey Gene No	Turkey Gene Nomemclature
**VIMP SELS**	NM_001024734.2	Gallus gallus VCP-interacting membrane protein (VIMP), mRNA	NC_015022.2	Meleagris gallopavo isolate NT-WF06-2002-E0010 breed Aviagen turkey brand Nicholas breeding stock chromosome 12, Turkey_5.0, whole genome shotgun sequence
**SELT LOC100548711**	NM_001006557.3	Gallus gallus selenoprotein T (SELT), mRNA	NC_015021.2	Meleagris gallopavo isolate NT-WF06-2002-E0010 breed Aviagen turkey brand Nicholas breeding stock chromosome 11, Turkey_5.0, whole genome shotgun sequence
**SELU FAM213A**	NM_001193518.1, I	Gallus gallus chromosome 6 open reading frame, human C10orf58 (C10ORF58), transcript variant 1, mRNA, Gallus gallus chromosome 6 open reading frame, human C10orf58 (C6H10orf58), transcript variant 2, mRNA	NC_015018.2	Meleagris gallopavo isolate NT-WF06-2002-E0010 breed Aviagen turkey brand Nicholas breeding stock chromosome 8, Turkey_5.0, whole genome shotgun sequence
**MSRB1 SEPX1**	NM_001135558.2	Gallus gallus methionine sulfoxide reductase B1 (MSRB1), mRNA	NC_015026.2	Meleagris gallopavo isolate NT-WF06-2002-E0010 breed Aviagen turkey brand Nicholas breeding stock chromosome 16, Turkey_5.0, whole genome shotgun sequence
**SEP15**	NM_001012926.2	Gallus gallus 15 kDa selenoprotein (SEP15), mRNA	NC_015020.2	Meleagris gallopavo isolate NT-WF06-2002-E0010 breed Aviagen turkey brand Nicholas breeding stock chromosome 10, Turkey_5.0, whole genome shotgun sequence
**SEPN1**	NM_001114972.1	Gallus gallus selenoprotein N, 1 (SEPN1), mRNA	NC_015035.2	Meleagris gallopavo isolate NT-WF06-2002-E0010 breed Aviagen turkey brand Nicholas breeding stock chromosome 25, Turkey_5.0, whole genome shotgun sequence

Compiled as of February, 2015

S2 Table. NCBI Reference Sequences and Nomenclature for Chicken Selenoprotein Transcripts and Turkey Selenoprotein Genes, page 1

Gene	Chicken Transcript No	Chicken Transcript Nomemclature	Turkey Gene No	Turkey Gene Nomemclature
**SEPP1**	NM_001031609.2	Gallus gallus selenoprotein P, plasma, 1 (SEPP1), mRNA	NC_015041.2	Meleagris gallopavo isolate NT-WF06-2002-E0010 breed Aviagen turkey brand Nicholas breeding stock chromosome Z, Turkey_5.0, whole genome shotgun sequence
**SEPP2**			NC_015020.2	Meleagris gallopavo isolate NT-WF06-2002-E0010 breed Aviagen turkey brand Nicholas breeding stock chromosome 10, Turkey_5.0, whole genome shotgun sequence
**SEPW1**	NM_001166327.1	Gallus gallus selenoprotein W, 1 (SEPW1), mRNA		
**TXNRD1**	NM_001030762.2	Gallus gallus thioredoxin reductase 1 (TXNRD1), mRNA	NC_015011.2	Meleagris gallopavo isolate NT-WF06-2002-E0010 breed Aviagen turkey brand Nicholas breeding stock chromosome 1, Turkey_5.0, whole genome shotgun sequence
**TXNRD2**	NM_001122691.1	Gallus gallus thioredoxin reductase 2 (TXNRD2), nuclear gene encoding mitochondrial protein, mRNA	NC_015027.2	Meleagris gallopavo isolate NT-WF06-2002-E0010 breed Aviagen turkey brand Nicholas breeding stock chromosome 17, Turkey_5.0, whole genome shotgun sequence
**TXNRD3**	NM_001122777.1	Gallus gallus thioredoxin reductase 3 (TXNRD3), mRNA	NC_015024.2	Meleagris gallopavo isolate NT-WF06-2002-E0010 breed Aviagen turkey brand Nicholas breeding stock chromosome 14, Turkey_5.0, whole genome shotgun sequence
**SEPHS1**	NM_001164084.1	Gallus gallus selenophosphate synthetase 1 (SEPHS1), mRNA	NC_015011.2	Meleagris gallopavo isolate NT-WF06-2002-E0010 breed Aviagen turkey brand Nicholas breeding stock chromosome 1, Turkey_5.0, whole genome shotgun sequence

Compiled as of February, 2015
