# Supplementary material for: Cloning, Sequencing, and Expression of Selenoprotein Transcripts in the Turkey (Meleagris gallopavo)
Source: PLoS One. 2015 Jun 12;10(6):e0129801. doi: 10.1371/journal.pone.0129801 (PMC4466519; doi:10.1371/journal.pone.0129801)
Supplement: S3 Table — For each selenoprotein, transcript coding sequences (CDS) in the transcript sequences reported here were compared with coding sequences for chicken (Gallus gallus), human (Homo sapiens), and mouse (Mus musculus) using NCBI Blast software (http://blast.ncbi.nlm.nih.gov/Blast.cgi), to determine sequence identity. (PDF) [file pone.0129801.s003.pdf]

S3 Table. Sequence Identity of Turkey Selenoprotein Transcripts relative to Chicken, Human, and Mouse sequences

		CDS	Chicken (Gallus gallus)		Human (Homo sapiens)		Mouse (Mus musculus)	
Gene	Alias	bp	%[1]	Sequence No.	%[1]	Sequence No.	%[1]	Sequence No.
GPX1		588	97	NM_001277853.1	78.5[2]	NM_000581.2	75	NM_008160.6
GPX2		576	97	NM_001277854.1	75	NM_002083.3	74	NM_030677.2
GPX3		660	98	NM_001163232.2	74	NM_002084.3	74	NM_008161.3
GPX4		579	95	AF498316.2	76.5[2]	NM_001039848.1	75	NM_001037741.2
DIO1		741	95	NM_001097614.1	69	NM_000792.5	69	NM_007860.3
DIO2		1213	94	NM_204114.3	78.5[2]	NM_001242503.1	78	NM_010050.2
DIO3		772	99	NM_001122648.1	76	NM_001242503.1	69	NM_172119.2
SELH	C5H11orf31	178	97	NM_001277865.1	73	NM_170746.2	67	NM_001037279.1
EPT1	SELI	631	97	NM_001031528.2	72	NM_033505.2	72	NM_027652.2
SELK	LOC100544511	288	97	NM_001025441.2	74	NM_021237.3	71	NM_019979.2
SELM	LOC100546114	395	97	NM_001277859.1	77	NM_080430.2	75	NM_053267.2
SELO	LOC100539640	1470	96	NM_001115017.1	70	NM_031454.1	71	NM_027905.2
VIMP	SELS	132	96	NM_001024734.2	79	NM_203472.2	79	NM_024439.3
SELT	LOC100548711	281	99	NM_001006557.3	91	NM_016275.3	90	NM_001040396.2
SELU	FAM213A	690	96	NM_001193518.1	73	NM_032333.4	71	NM_027464.3
MSRB1	SEPX1	507[3]	92	NM_001135558.2		none[3]		none[3]
SEP15		483	97	NM_001012926.2	76	NM_004261.3	82	NM_053102.2
SEPN1		955	98	NM_001114972.1	80	NM_206926.1	78	NM_029100.2
SEPP1		1000	95	NM_001031609.2	79	NM_005410.2	74	NM_009155.3
SEPP2		329	95	XM_003641687.2		None[4]		None[4]
SEPW1		258	96	NM_001166327.1	67	NM_003009.2	67	NM_009156.2
TXNRD1		1027	97	NM_001030762.2	80	NM_003330.3	76	NM_001042523.1
TXNRD2		1354	95	NM_001122691.1	70	NM_006440.4	71	NM_013711.3
TXNRD3		503[3]	93	NM_001122777.1		none[3]		none[3]
SEPHS1		1179	98	NM_001164084.1	82	NM_012247.4	82	NM_175400.6

[1]Sequence identity, using the turkey transcript coding sequence (CDS) from the present study, was determined using NCBI BLAST (http://blast.ncbi.nlm.nih.gov/Blast.cgi) against the *Gallus gallus* (taxid:9031), *Homo sapiens* (taxid:9606), and *Mus musculus* (taxid:10090) databases

[2]Non-integer values are the mean sequence identity for transcripts with multiple human variants

[3]For cloned transcripts that only contained 3'UTR sequences, identities were determined using the 3'UTR sequence only against the chicken database.

[4]SEPP2 gene/transcript not found in the human or mouse.
